# Markets and the crowding out of conservation‐relevant behavior

**DOI:** 10.1111/cobi.13606

**Published:** 2020-10-08

**Authors:** Joshua E. Cinner, Michele L. Barnes, Georgina G. Gurney, Stewart Lockie, Cristian Rojas

**Affiliations:** ^1^ ARC Centre of Excellence for Coral Reef Studies James Cook University Townsville QLD 4811 Australia; ^2^ Cairns Institute James Cook University Cairns QLD Australia

**Keywords:** collective action, crowd out, equity, externalities, proenvironment behavior, acción colectiva, comportamiento proambiental, desplazamiento, efectos, externos, equidad, 亲自然行为, 排挤效应, 集体行动, 公平, 外部性

## Abstract

Markets are increasingly being incorporated into many aspects of daily life and are becoming an important part of the conservation solution space. Although market‐based solutions to environmental problems can result in improvements to conservation, a body of social science research highlights how markets may also have unforeseen consequences by crowding out or displacing 3 key types of behaviors potentially relevant to conservation, including people's willingness to engage in collective action and civic duty; tolerance for inflicting harm on others (third‐party externalities); and desire for equity. Better understanding of the contexts and mechanisms through which this crowding out occurs and whether specific market‐based instruments are more prone to different types of crowding out will be crucial to developing novel conservation initiatives that can reduce or prevent crowding out.

## Abstract 2

*Article impact statement*: Markets are important to conservation‐solution space but can crowd out 3 aspects of human behavior relevant for conservation.

## Abstract (es)

Los Mercados y el Desplazamiento del Comportamiento Relevante para la Conservación

**Resumen**

Los mercados cada vez están más incorporados dentro de muchos aspectos de la vida diaria y se están transformando en una parte importante del espacio de las soluciones de conservación. Aunque las soluciones basadas en los mercados para los problemas ambientales pueden resultar en mejoras para la conservación, una parte de los estudios sociales resaltan cómo los mercados también pueden tener consecuencias imprevistas al desplazar o excluir tres tipos importantes de comportamiento potencialmente relevantes para la conservación: la disposición de las personas a participar en acciones colectivas y deberes cívicos, la tolerancia a infligir daño a otros (efectos externos de terceros) y el anhelo por la equidad. Un mejor entendimiento de los contextos y los mecanismos mediante los cuales ocurre este desplazamiento y si los instrumentos basados en los mercados son más susceptibles a los diferentes tipos de desplazamiento serán elementos cruciales para desarrollar iniciativas de conservación novedosas que puedan reducir o prevenir el desplazamiento.

## 摘要

市场正越来越多地融入日常生活的方方面面, 并成为保护解决方案的重要组成部分。虽然以市场为基础的环境问题解决方案可能会有助于保护, 但大量社会科学研究也强调了市场可能通过排挤或取代与保护潜在相关的三种关键行为产生不可预见的后果, 这三种行为包括人们参与集体行动和履行公民义务的意愿、对于伤害他人的容忍(第三方外部性), 以及对公平的渴望。更好地理解这种排挤效应发生的背景和机制, 以及基于市场的特定工具是否更容易导致不同类型的排挤, 对于发展新的保护措施以减少或防止排挤, 将是至关重要的。**【翻译: 胡怡思; 审校: 聂永刚】**

## Introduction

Markets play a critical role in the mediation of people's relationship with nature through their influence on social (Polanyi & MacIver 1944; King & Pearce 2010) and environmental change (York et al. 2003). Markets facilitate the exchange of goods and services by providing for the transfer of information, the setting of prices, and the space (physical and virtual) in which exchange takes place (Sandel 2012). Infrastructure developments, such as China's Belt and Roads initiative, are providing more people with physical access to global markets (Laurance & Arrea 2017), and phone and internet connections are extending digital market access to ever more remote areas (Donner & Escobari 2010). Additionally, governments are using market (i.e., neoliberal) logic to guide policy in areas as diverse as trade, social welfare, and, importantly, environmental management.

This expansion of markets has major implications for conservation (Laurance & Arrea 2017). On the one hand, connections to markets can create incentives to increase resource exploitation and intensify land use (Boserup 1965; Lambin et al. 2001; Schmitt & Kramer 2009; Eakin et al. 2014). On the other hand, markets are becoming an important part of the conservation solution space. Market‐based instruments (Table 1), such as sustainability certifications for seafood, agriculture, and forestry products (Sampson et al. 2015), aim to improve the social and environmental performance of existing markets by increasing the flow of information between producers and consumers and providing a means through which the costs of environmental care can be passed on (Lockie 2020). Other market‐based instruments create new markets in the provision of environmental goods or in the avoidance of environmental harm. These include individually transferrable quotas or rights to access natural resources (Costello et al. 2008), tradable rights in biodiversity and pollution offsets (Bull et al. 2013; Ferreira & Ferreira 2019; Lockie 2020), and payments for ecosystem services (Farley & Costanza 2010; Ramsdell et al. 2016) (Table 1). In New Zealand's Lake Taupo catchment, for example, the introduction of a water‐quality trading scheme is lowering the cost to farmers of reducing nitrogen pollution and shifting land use toward activities that produce more economic value for each unit of nitrogen discharged (Duhon et al. 2015). Other work highlights both benefits and perverse outcomes that can arise from ecotourism, which markets and consumes not only species and ecosystems, but also communities and their cultural traditions as a means to conserve them (Duffy 2008; Stronza et al. 2019).

**Table 1:** Typology of market‐based conservation instruments.*

Classification	Market intervention goal	Situation for application	Action	Examples
Market friction	Remove obstacles to recognition of natural resource inputs in existing markets.	Outcomes can be improved through increased information.	standards	sustainable production and harvest standards (e.g., Marine Stewardship Council); management system standards (e.g., ISO14001 Environmental Management Systems) (Bush et al. 2013; Lockie 2020)
			auditing and verification	third‐party certification schemes (e.g., MSC Certified)
			communication	ecolabels (e.g., MSC Blue Tick)
Market reform	Set or modify prices to incorporate the cost of environmental protection.	Pollutant emissions and resource extractions are measureable.	environmental levies	Protected‐area visitor charges (Farr et al. 2011)
			ecotaxes	pesticide taxes, carbon taxes (Böcker & Finger 2016)
Quantity‐based markets	Establish market mechanisms to reallocate resources within set emission or extraction targets.	Pollutant emissions and resource extractions are measureable.	tradable emission or extraction rights	tradeable fisheries quotas; tradeable water rights (Bigger 2018)
			tradable offsets	water‐quality trading credits; biodiversity offsets; carbon offsets (Woodward et al. 2016)
			cap‐and‐trade mechanisms	greenhouse gas emissions trading systems; pollution trading (Ranson & Stavins 2016)
Market design	Utilize market mechanisms to allocate payments for ecosystem service provision.	Multiple resource users can provide improved environmental outcomes.	conservation tenders or reverse auctions	biodiversity auctions (Tennent and Lockie 2013)
Other financial incentives	Allocate investment to targeted resource users through nonmarket means.	Environmental outcomes require involvement of all resource users.	direct payments	conservation subsidies; Environmental cross‐compliance requirements (Claassen et al. 2013)
			tax incentives	tax credits or rebates for resource conservation; property tax waivers for conservation (Kerr & Winskel 2020)
Property right mechanisms	Establish rights that enable market exchange or place agreed restrictions on future use.	Market incentives or private investment will be facilitated by clearly defined property rights and responsibilities.	voluntary agreements to manage private land for conservation	private land trusts; conservation easements (Parker & Thurman 2019)
			private management of protected areas	conservation concessions; ecotourism concessions (Schleicher et al. 2017)

* Adapted from Lockie (2013).

Through their expansion, in both scale and scope, markets are not only connecting more people across greater expanses of space, but also infiltrating more areas of people's private and social lives and becoming part of cultures and institutions. Sandel (2012) notes, “we have drifted from *having* a market economy, to *being* a market society” in which activities that were previously governed by non‐market values are now commodified, including paying for school children to read, people to wait in lines, the right to drive solo in carpool lanes, and even the sterilization of drug addicts. As societies embrace neoliberalism and drift toward being market societies, the assumptions of markets are often accepted uncritically. Consequently, it is sometimes difficult to recognize and carefully examine the potentially nefarious and long‐term consequences of such a societal shift (Sandel 2012). We considered one of these consequences and its relevance to conservation: how markets and market‐based instruments may displace or “crowd out” (Gneezy & Rustichini 2000a; Sandel 2012; Falk & Szech 2013) behaviors potentially relevant to conservation and lead to unforeseen or perverse outcomes (Reddy et al. 2017). More specifically, we focused on how expanding engagement with markets may crowd out behaviors related to three key areas relevant to conservation: willingness to engage in collective action and civic duty; tolerance for inflicting harm on others (third‐party externalities); and desire for equity. Our goals were to bring the important body of work on markets and crowding out to the attention of the broader audience of conservation scientists, highlight the implications for conservation, and put forward a research agenda that can help in the design and implementation of conservation initiatives that reduce or avoid crowding out from markets.

### Crowding Out

Crowding out is a well‐established phenomenon in economics, social psychology, political science, and environmental sociology (e.g., Frey & Jegen 2001; Agrawal et al. 2015; Lockie 2020). Initially used to describe how government spending programs reduce investments in the private sector, the concept of crowding out has been associated with the displacement of motivation for more than two decades (Frey 1997). Crowding theory is underpinned by the idea that motivation arises from both extrinsic and intrinsic sources (Deci 1971, 1975). Extrinsic motivation refers to behavior that is driven by external rewards, such as money or praise. In contrast, intrinsic motivation relates to undertaking an activity or behavior for the inherent satisfaction it brings (Young 1986). The basic notion of crowding out is that extrinsic motivators (such as spending programs, payments, prices) can displace people's intrinsic motivation to engage in certain behaviors (Frey & Jegen 2001; Gneezy et al. 2011; Rode et al. 2015). Extrinsic motivators can also crowd in (reinforce) intrinsic motivation (Lazear 2000; Duflo et al. 2012; Acland & Levy 2015).

Crowding out has been highlighted as a perverse outcome from a broad range of public policy domains, including blood donation (Titmuss 1970), charitable fundraising efforts (Gneezy & Rustichini 2000b), workplace motivation (Glewwe et al. 2010), and child care (Gneezy & Rustichini 2000a). For example, many day care facilities have a problem with parents being late to pick up their children. In Israel, some day care centers attempted to reduce tardy pickups by imposing a fine for being late (Gneezy & Rustichini 2000a). In response, the incidence of late pickups nearly doubled. What happened? Prior to the fines, social norms made parents feel bad for picking up their children late, but the fines created the idea of compensation for the extra time, displacing the ethical obligation to be punctual. Three weeks later, when the fines were reversed, the elevated rate of late pickups persisted (Gneezy & Rustichini 2000a). Once eroded, the moral obligation to be on time was hard to revive (i.e., it had been crowded out) (Reeson & Tisdell 2008; Yasué et al. 2019).

The ways that external incentives can crowd out conservation‐relevant behavior has been widely investigated in a range of contexts (Rode et al. 2015), including cooperation (Cardenas et al. 2000), protected areas (Cetas & Yasué 2017), recycling (Young 1986; Feldman & Perez 2012), and energy use (Pellerano et al. 2017). For example, in Colombia a series of experiments designed to examine the effect of regulations on environmental quality revealed that certain regulations had the perverse outcome of crowding out group‐oriented decisions with self‐oriented decisions that resulted in participants receiving lower earnings (Cardenas et al. 2000). Likewise, in Indonesia, material incentives provided by a USAID‐funded integrated conservation and development project were suggested to have crowded out people's intrinsic incentives to participate in marine management by reframing management as an externally driven activity rather than a community activity governed by customary social norms (Gurney et al. 2016). However, our focus—the potential for markets to crowd out potentially relevant conservation behavior—has only recently gained traction among the conservation community (Rojas & Cinner 2020), primarily in the domain of payments for ecosystem services (Akers & Yasué 2019; Ezzine‐de‐Blas et al. 2019; Kaczan et al. 2019). A substantial body of work shows that engagement in payment for ecosystem service markets can crowd out people's intrinsic motivations to engage in conservation (Rico García‐Amado et al. 2013; Akers & Yasué 2019; Ezzine‐de‐Blas et al. 2019) and in some cases may even fundamentally change people's relationship with nature by crowding out subsistence values with market‐oriented values (Chervier et al. 2019). For example, in Cambodia the introduction of a payments for ecosystem services scheme shifted people's perceived forest values from being primarily subsistence related (i.e., for food security, shelter, and health) to primarily money related (Chervier et al. 2019). We built on this work by highlighting three additional ways that markets can crowd out key behaviors and preferences that are relevant to conservation more broadly.

### Markets and the Crowding Out of Conservation‐Relevant Behavior

**Figure 1:**
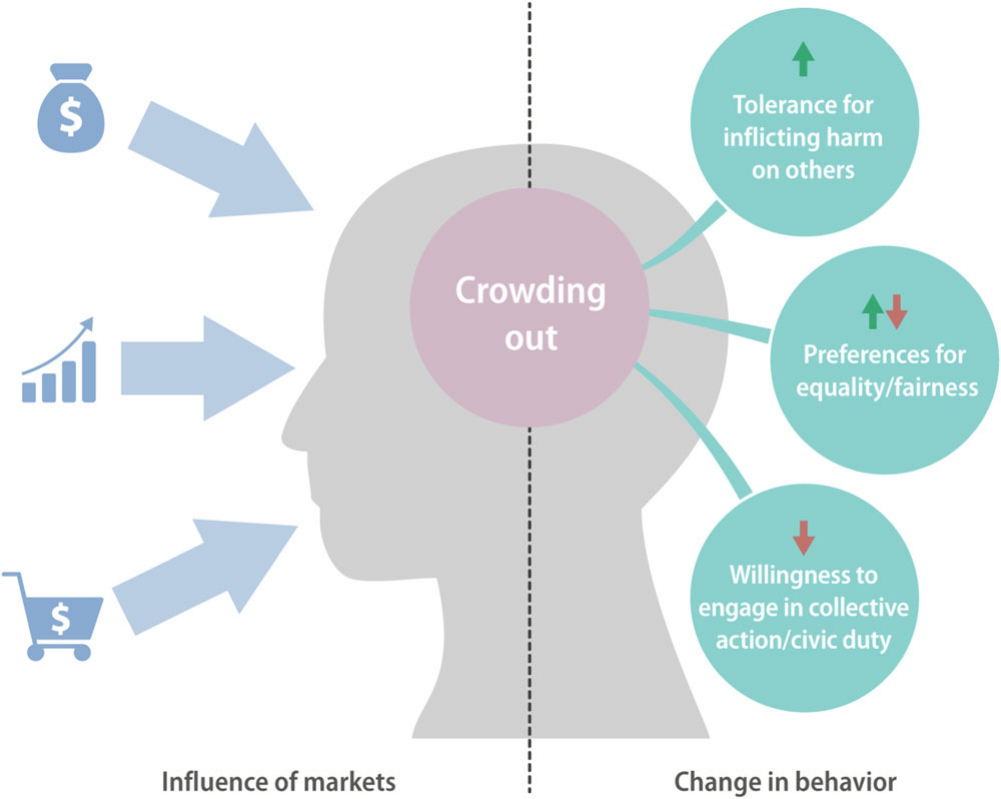
Market forms (left), ranging from physical places of exchange to the commodification of everyday behaviors (e.g., paying children to read), that crowd out aspects of human behavior that may be relevant to conservation (small arrows, directionality of change due to markets according to the literature; red and green arrows, directionality of influence).

One of the most important ways that markets can crowd out potentially conservation‐relevant behavior is by reducing people's propensity to engage in collective action or civic duties (Fig. 1) (Gneezy and Rustichini 2000b). For example, in Australia, the use of market‐based incentives for rural land conservation has been associated with declining participation in community‐based natural resource management programs that rely on voluntary cooperation (Tennent & Lockie 2013). Critically, many community‐based approaches to conservation and sustainability rely on voluntary collective action and civic duty norms (Ostrom 1990, 2000) and may be vulnerable to this type of crowding out. Yet, it remains unknown the degree to which people consider certain types of sustainability‐relevant practices and behaviors to be civic duties (such as customary and traditional management (Cinner & Aswani 2007) or biodiversity and cultural heritage conservation (Hodge & Reader 2010) and may thus be vulnerable to crowding out by markets (Cinner et al. 2007).

Emerging research suggests markets can also affect people's willingness to inflict indirect harm on others, often referred to as negative or third‐party externalities (Falk & Szech 2013; Collins et al. 2018). The evidence demonstrating a link between the influence of markets on people's willingness to inflict externalities stems from controlled laboratory experiments in which market conditions are emulated, but to date, none are from a conservation context. For example, when given the choice between saving the life of an exlaboratory mouse bound for destruction or receiving a cash payment, participants were more willing to inflict an externality (in this case, the death of the mouse) under market conditions (i.e., where participants could bargain about the price and when there were multiple potential buyers or sellers) than under nonmarket conditions (Falk & Szech 2013). In another experiment that measured the production and consumption of products that were unfair (i.e., they imposed a monetary cost externality on a third party) and fair (i.e., no externality), people's willingness to inflict externalities (i.e., trade the unfair product) was higher under market conditions when compared with nonmarket conditions (Bartling et al. 2015). Failing to account for increased tolerance of externalities in a conservation context could mean, for example in fisheries, an increase in the use of destructive gear and fishing practices that compromise long‐term sustainability (Cho 2009). However, the body of research on market influences over people's preferences for inflicting externalities is still in its early stages, not all the experimental evidence is conclusive (Kirchler et al. 2016), and questions remain about whether the results from controlled experiments carry over into real life (i.e., external validity).

Markets can also affect people's preferences for social equity, or fairness (Bowles 1998). Evidence from a number of studies employing experimental economic games suggests that the presence of markets lowers fair‐minded behavior. For example, divisions were less equal under a market treatment when a dictator game (a 2‐player game where 1 player divides a monetary allocation between themselves and a passive recipient) was used to examine how behavior was affected when the right to decide the division was assigned versus determined in a market treatment (Collins et al. 2018). Likewise, reframing an ultimatum game (similar to dictator game, but where the recipient choses to accept or reject the proposed distribution, with the latter choice resulting in no payoff for either player) as a market game with sellers and buyers resulted in players allocating money less equitably (Hoffman et al. 1994). However, contrary to these studies, a seminal study of 15 small‐scale societies in 12 countries found that real‐life market integration was positively related to fair‐minded behavior (more equal divisions) in 3 types of games (Henrich et al. 2010). The authors suggest that market‐integrated societies have had to develop prosocial norms for dealing with strangers to sustain mutually beneficial exchanges in market situations where established social relationships (e.g., reciprocity, kin) were insufficient (Henrich et al. 2010). Therefore, while it is clear that markets affect preferences for fairness, further research is need to examine under what conditions markets lead to less or more fair‐minded behavior.

An additional aspect of equity that markets may also influence is people's preferences about what actually constitutes fairness. A promising line of inquiry is untangling whether people's perceived fairness in monetary distributions (i.e., distributional equity) actually manifests as equality, as assumed in many economic games (Starmans et al. 2017). Indeed, what is perceived to constitute a fair distribution of resources or burdens can follow a number of different principles (e.g., that relate to merit, need [Deutsch 1975]). Understanding how market integration influences preferences for specific distributional principles is limited, but emerging evidence suggests that switching from non‐monetary to monetary benefits is associated with changes in preferences for distributional fairness in ways that may be detrimental to the poor (Martin et al. 2019). A study of the influence of market‐based forestry interventions (e.g., sales of certified timbers and carbon credits) found that forest commodification was associated with less support for egalitarian approaches or approaches that benefit the poor than for approaches that rewarded individual contributions or compensated losses (Martin et al. 2019). Building understanding of how market integration affects preferences for specific distributional principles in the context of conservation is critical. Fairness is a key component of well‐being (Prilleltensky 2013), and perceived unfairness and the associated reduction in social capital (Pretty & Smith 2004) can reduce support for environmental management and conservation initiatives (Gurney et al. 2014) and undermine collective action on which many conservation approaches predicated (Tyler 1975).

### Toward A Research Agenda on Crowding Out in Conservation

A key question that remains is whether and how conservation initiatives can prevent the potential displacement of collective action, equity, and intolerance for externalities by markets? We suggest that answering this question will require a novel research agenda with three key foci. The first is testing the mechanisms underlying crowding out. A range of psychological mechanisms that can result in crowding out have been suggested, such as frame shifting, release from moral responsibility, reduced internal satisfaction, and “control aversion,” whereby a reduced sense of agency motivates resistance (Rode et al. 2015; Bowles & Polanía‐Reyes 2012). A key proposed mechanism through which incentives may lead to crowding out (or in) is via a shift in the social norms regarding the behavior in question (Göckeritz et al. 2010; Bowles & Polanía‐Reyes 2012). For example, Kerr et al. (2019) examined the role of descriptive norms (perceptions of the prevalence of the behavior) and injunctive norms (perceptions of others’ approval of the behavior) in motivational crowding with regards to payments for participating in conservation enforcement patrols in Nepal. They found that the incentive heightened a perceived injunctive norm that the conservation behavior met with social approval, thus leading to crowding in.

Further, existing research also suggests that the degree of crowding out can vary depending on the types of motivations people have for engaging in prosocial or pro‐environmental behavior (Ariely et al. 2009). For example, external incentives can crowd out what is referred to as “image motivation” (i.e., engaging in prosocial behavior to improve ones’ social image) (Ariely et al. 2009). A review of experimental literature suggests prosocial behavior may be influenced by preferences for appearing to be fair (i.e., social image) rather than preferences for actual fairness (Collins et al. 2018). In a conservation context, Australian farmers participating in reverse auctions for biodiversity conservation had mixed feelings about the receipt of public money to protect native vegetation (Tennent & Lockie 2013). While some valued this incentive, others thought it undermined their public reputation as good stewards of the landscape and led to little or no conservation activity beyond what would have been undertaken. These programs led to concern that farmers’ duty to provide environmental care was being undermined (Lockie 2013, 2020).

The second key avenue for future research is identifying the contexts under which crowding out of conservation‐relevant behavior may be more or less likely. For example, crowding out has been shown to be more likely when external implementing agencies are perceived as controlling rather than supportive and when existing norms of reciprocity and cooperation are strong (Vollan 2008; Gurney et al. 2016). In regards to the latter, where initial levels of cooperation and reciprocity are low, interventions can perform well in encouraging desired behaviors and are unlikely to lead to crowding out because, quite simply, there is no cooperative behavior to be crowded out. Systematically investigating the contexts that enable or inhibit crowding out will require building off of theories (Ryan & Deci 2000) and frameworks (Ezzine‐de‐Blas et al. 2019) designed to investigate crowding out (Cetas & Yasué 2017; Akers & Yasué 2019). Such investigations may include interrogating relevant psychological needs (competence, autonomy, social relatedness, and environmental relatedness); personal context of resource users (e.g., levels of education, wealth, and culture); interpersonal context (e.g., social norms and institutions); policy context (e.g., whether different types of market‐based instruments (Table 1) are prone to specific forms of crowding out); implementation context (e.g., whether the implementing agency is government, NGO, private sector, and how they operate); decision context (e.g., whether behaviors are one‐off or repeated, made under high or low uncertainty, visible or discrete); resource access (e.g., club, private, public good, or common pool resource); and how resource users justify why they engage in certain behaviors. Indeed, this line of research may uncover when crowding out presents trade‐offs regarding the promotion or reduction of desirable and undesirable behaviors (Cetas & Yasué 2017; Chamberlin et al. 2018).

Finally, testing whether conservation initiatives can be coupled with countermeasures to prevent or reduce crowding out is a further fruitful area for future research. Examples include coupling conservation initiatives with measures that foster intrinsic motivations or reinforce people's moral responsibility, recognizing multiple stakeholders may hold heterogenous motivations. For example, research on early childhood education suggests that intrinsic motivation can be fostered through supporting autonomy or agency, strong social bonds or capital, self‐evaluation, and limited external rewards (Carlton & Winsler 1998). Fieldwork, lab experiments, and lab‐in‐the‐field experiments will be necessary to rigorously test how markets may crowd out certain behaviors—or alter the motivation for these behaviors (i.e., shift from image motivation to external motivation)—and the contexts under which this can happen. Alternatively, rigorous impact analysis will be required to test the outcomes of coupling conservation initiatives with countermeasures.

## Conclusion

Economic orthodoxy suggests that properly functioning markets provide incentives for the efficient use of natural resources (Stavins 2003). Examples abound of increased resource extraction being incentivized by market failures, which occur when the long‐term impacts of particular resource‐use activities are not well understood, property rights are insecure or absent, natural resources are priced below their full environmental and economic value, or when producers are unable to pass these costs on to their customers (York et al. 2003; Schmitt & Kramer 2009; Stevens et al. 2014; Lockie 2020). Market‐based instruments have been developed to help correct these types of market failures (Table 1), and numerous examples can also be found of resource management practices that have improved following their introduction (Costello et al. 2008). However, market‐based approaches in societal sectors ranging from education, to health care, to justice (i.e., incarceration) have had unforeseen outcomes, and conservation is no different. We highlighted an emerging field of research that points to the potential for markets to crowd out collective action, preferences for equity, and intolerance of externalities– a topic beyond the scope of traditional market failure and one that current market‐based instruments are ill‐prepared for and may actually exacerbate. Our purpose was not to discourage those that use market‐based instruments to address environmental problems, but rather to highlight and catalyze discussion about this area of emerging research that may have profound relevance to conservation. Indeed, such discussions may be necessary for these market‐based solutions to reach their full conservation potential and achieve long‐term behavior change. In addition to the use of market‐based initiatives, the crowding out effects discussed here may also have relevance for conservation initiatives designed to promote alternative market‐based livelihoods (e.g. trophy hunting) and for infrastructure development projects that could increase market integration.
